# Effectiveness of Best Management Practices to Reduce Phosphorus Loading to a Highly Eutrophic Lake

**DOI:** 10.3390/ijerph15102111

**Published:** 2018-09-25

**Authors:** Alan D. Steinman, Michael Hassett, Maggie Oudsema

**Affiliations:** Annis Water Resources Institute, Grand Valley State University, 740 W. Shoreline Drive, Muskegon, MI 49456, USA; hassetmi@gvsu.edu (M.H.); oudsemam@gvsu.edu (M.O.)

**Keywords:** best management practices, eutrophication, Lake Macatawa, watershed restoration

## Abstract

Reducing nonpoint source pollution is an ongoing challenge in watersheds throughout the world. Implementation of best management practices, both structural and nonstructural, is the usual response to this challenge, with the presumption that they are effective. However, monitoring of their efficacy is not a standard practice. In this study, we evaluate the effectiveness of two wetland restoration projects, designed to handle runoff during high flow events and serve as flow-through retention basins before returning flow further downstream. The Macatawa Watershed is located in west Michigan, is heavily agricultural, and drains into Lake Macatawa, a hypereutrophic lake with total phosphorus concentrations usually exceeding 100 µg/L. We measured turbidity, total phosphorus, and soluble reactive phosphorus both upstream and downstream of these wetland complexes during base flow and storm events. While both turbidity and phosphorus increased significantly during storm events compared to baseflow, we found no significant difference in upstream vs. downstream water quality two years following BMP construction. We also measured water quality in Lake Macatawa, and found the lake remained highly impaired. Possible reasons for the lack of improved water quality: (1) The restored wetlands are too young to function optimally in sediment and phosphorus retention; (2) the scale of these BMPs is too small given the overall loads; (3) the locations of these BMPs are not optimal in terms of pollutant reduction; and (4) the years following postconstruction were relatively dry so the wetlands had limited opportunity to retain pollutants. These possibilities are evaluated.

## 1. Introduction

Eutrophication of freshwater ecosystems is a global problem, resulting in both ecological and societal challenges, including potentially harmful algal blooms, hypoxia, loss of biodiversity, and economic impacts to home value and ecotourism [1,2,3,4]. These challenges are particularly problematic in heavily agricultural systems, where runoff can carry significant loads of sediment and nutrients, resulting in legacy issues in terrestrial soils and lake sediments [5].

Another challenge is identifying which conservation practices (or best management practices: BMPs) are most appropriate and where are the most desirable locations to place them, in order to use limited resources most effectively to achieve restoration goals [6]. In other words, as provocatively asked by Sowa et al. [7], how much conservation is enough?

One of the most significantly impaired lakes in Michigan is Lake Macatawa, which is the terminus of a highly degraded watershed and has exhibited the symptoms of a hypereutrophic lake for more than 40 years [8,9]. Extremely high nutrient and chlorophyll concentrations, excessive turbidity, low dissolved oxygen, and a high rate of sediment deposition make it one of the most hypereutrophic lakes in Michigan [8,9]. Nonpoint source pollution from the watershed, particularly agricultural areas, is recognized as the primary source of the excess nutrients and sediment that fuel hypereutrophic conditions in Lake Macatawa [8].

Because of this nutrient enrichment, Lake Macatawa and all of its tributaries are included on Michigan’s list of impaired water bodies, prompting the issuance of a phosphorus Total Maximum Daily Load (TMDL) for Lake Macatawa in 2000. The TMDL set an interim target total phosphorus (TP) concentration of 50 μg/L in Lake Macatawa [10]. In recent years, monthly average TP concentrations were greater than 125 μg/L, and at times exceeded 200 μg/L [8]. Thus, meeting the TMDL target represents a major challenge in the Macatawa watershed. The TMDL estimated that a 72% reduction in phosphorus loads from the watershed would be required to meet the TP concentration target [10].

A large-scale, multidisciplinary, collaborative watershed remediation project aimed at improving water quality in Lake Macatawa was initiated in 2013 and called “Project Clarity”. This public–private partnership adopted a holistic approach that includes wetland restoration, in-stream remediation, BMPs, and community education, with the ultimate goal of improved water quality in Lake Macatawa.

In this paper, we analyze pre- and postrestoration water quality data of two wetland restoration projects, as well as lake status, to evaluate the performance of these BMPs, as well as overall watershed condition. We also speculate on why water quality has not significantly improved in the two years following construction of the restored wetlands.

## 2. Methods

### 2.1. Overall Site Description

The Macatawa watershed (464 km^2^) is located in Ottawa and Allegan Counties (MI) and includes Lake Macatawa, the Macatawa River, and many tributaries. It is dominated by agricultural (46%) and urban (33%) land uses, which have contributed to the loss of 86% of the watershed’s natural wetlands [8]. The watershed includes the cities of Holland and Zeeland and parts of 13 townships [8]. Lake Macatawa is a 7.2 km^2^ drowned river mouth lake. It is relatively shallow, with an average depth of 3.6 m and a maximum depth of 12 m in the western basin. The Macatawa River, the main tributary to the lake, flows into the lake’s shallow eastern basin. A navigation channel at the western end of the lake connects Lake Macatawa with Lake Michigan.

**Figure 1:**
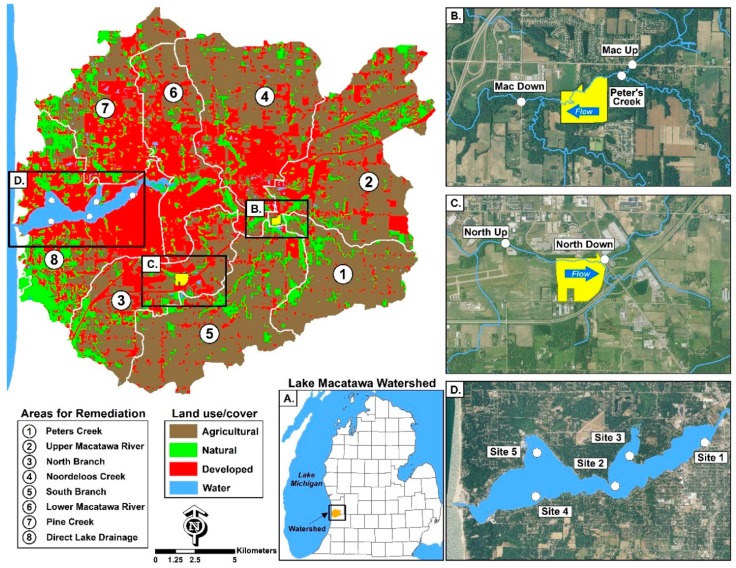
Land use/land cover in the Macatawa watershed, divided into the eight major sub-basins (separated by white lines). Box (**A**) (lower center): location of Lake Macatawa Watershed within the lower peninsula of Michigan. Box (**B**) (upper right): Middle Macatawa wetland restoration area (footprint in yellow) with the two upstream and one downstream sampling locations. Box (**C**): Haworth wetland restoration area (footprint in yellow) with the upstream and downstream sampling locations. Box (**D**): Lake Macatawa showing the five sampling locations (white dots) for water quality monitoring.

A watershed-wide sediment sampling study, conducted in 2011 and 2012 (Hope College, Holland, MI, USA, unpubl. data) identified the sub-basins within the Macatawa watershed with the highest amounts of sediment. Based on these results, the Peters Creek, Upper Macatawa River, and North Branch sub-basins (Figure 1) were given the greatest priority for restoration action. As a consequence, two wetland restoration efforts were targeted for these regions: Middle Macatawa and Haworth projects. The specific sites were selected due to land owners’ willingness to donate or sell the parcels. Our monitoring initiative is focused on these two key wetland restoration areas in the Macatawa watershed and Lake Macatawa (Figure 1). Details on these two efforts are provided below.

### 2.2. Wetland Restoration: Middle Macatawa & Haworth Properties

The Middle Macatawa and Haworth properties were purchased as part of Project Clarity and designated for wetland restoration. Restoration goals included slowing the flow of water in the Macatawa River and its tributaries, particularly during high flow events, thus trapping and retaining suspended sediments and nutrients. The Middle Macatawa project involved reconnecting ~16 ha of former pastureland to the adjacent river by placing a pipe from the river to an excavated area in the floodplain. The spoils from the excavated area create a berm that surrounds the excavated area and detains floodwater, allowing sediment and nutrients to settle out. The Haworth project restored a 17-ha wetland, composed of four basins that collect and store water from adjacent properties and the river during periods of high flow. Restoration construction at Middle Macatawa and Haworth was completed in late September and early October 2015, respectively.

Monitoring sites were established upstream and downstream of each restoration area. The Middle Macatawa study area (Figure 1B) has two upstream sites (Macatawa River (Macatawa Up) and Peters Creek), which both flow into the Macatawa River and one downstream site (Macatawa River at the USGS gauging station (Macatawa Down)). The Haworth study area (Figure 1C) consists of monitoring locations upstream and downstream of the restoration area on the North Branch of the Macatawa River. Our focus in this paper is on water quality and hydrologic monitoring from December 2016 through November 2017, although we also include prior monitoring data. Sampling occurred monthly during baseflow conditions and during three storm events in 2017 (Table 1); storm sampling was triggered when the local USGS gage reached 300 ft^3^/s (~8.5 m^3^/s). During each monitoring event, general water quality parameters (dissolved oxygen (DO), temperature, pH, specific conductivity (SpCond), total dissolved solids (TDS), redox potential (ORP)) were measured using a YSI 6600 sonde. Grab samples were collected for analysis of phosphorus (soluble reactive phosphorus (SRP), TP) and nitrogen (ammonia (NH_3_), nitrate (${\mathrm{NO}}_{3}^{-}$), and total Kjeldahl nitrogen (TKN)) species. Water quality measurements and sample collection took place in the thalweg of the channel at permanently-established transects. Duplicate water quality samples and sonde measurements were taken every other month during baseflow conditions and all storm events. All samples were placed in a cooler on ice until returned to the lab, usually within four hours, where they were stored and processed appropriately (see below).

Water for SRP and ${\mathrm{NO}}_{3}^{-}$ analyses was syringe-filtered through acid-washed 0.45-μm membrane filters into scintillation vials; SRP was refrigerated and ${\mathrm{NO}}_{3}^{-}$ frozen until analysis. NH_3_ and TKN were acidified with sulfuric acid and kept at 20 °C until analysis. SRP, TP, NH_3_, ${\mathrm{NO}}_{3}^{-}$, and TKN were analyzed on a SEAL AQ2 discrete automated analyzer (SEAL Analytical, Inc.: Mequon, WI, USA) [11]. Any values below detection were calculated as ½ the detection limit.

Stream hydrographs were generated at each monitoring location using water level loggers and staff gages that were installed at permanently established transects at four of the monitoring locations (the Macatawa Down site did not require one because we used the USGS gage).

Turbidity sensors were deployed at the upstream and downstream locations on the main branch of the Macatawa River before snowmelt in March 2017. The in situ turbidity sensors are YSI 600OMS V2 (Xylem Inc.: Rye Brook, NY, USA), which uses the same technology as the YSI 6600 through the same YSI 6136 optical turbidity sensor. The 6136 turbidity sensor has a range of 1 to 1000 NTU with a resolution 0.1 NTU, and accuracy of ± 2% of the reading or 0.3 NTU, whichever is greater. It features a mechanical self-wiping capability to help prevent biofilm covering the optics. We placed the turbidity sensors in a protective housing made from schedule 80 PVC with vents at the bottom to allow water to pass through. The measurements are recorded every 30 min, and stored internally until downloaded once a month. The YSI sensors are calibrated on a monthly basis. The turbidity sensors were removed in December 2017 to avoid possible ice damage and were returned to their former locations before the final snowmelt in spring of 2018.

### 2.3. Data Analysis

Our analysis focused on identifying (1) upstream vs. downstream differences during baseflow and storm flow conditions, and (2) pre- vs. postrestoration differences in nutrients and turbidity. Upstream–downstream differences between site pairs (e.g., North Up vs. North Down) within the 2017 sampling year at baseflow and at storm flow were statistically tested using either a two-tailed paired *t*-test (normally-distributed data) or Wilcoxon signed rank test (non-normally distributed data). Baseflow and storm flow conditions were evaluated separately for each site pair. A one-way analysis of variance test (ANOVA; normally distributed data) or Kruskal–Wallis test (one-way ANOVA on ranks; non-normally distributed data) was used to compare data from the three Middle Macatawa sites simultaneously. ANOVAs that detected significant differences were followed by post-hoc Tukey pairwise comparison tests.

Pre- and postrestoration differences were statistically tested separately for each site using two-tailed paired *t*-tests at baseflow and either two-tailed unpaired *t*-tests (normally distributed data) or Mann–Whitney rank sum tests (non-normally distributed data) at storm flow. In order to remove seasonality as a potentially biasing factor in analyses and because not all samples were taken at the same time from all sites, paired *t*-tests for baseflow incorporated an equal number of samples (*n* = 16) from identical months in pre- and postrestoration periods (Apr., Jun., Jul., Sep., Oct., Nov., Dec., Jan., Feb., Mar., Apr., May, Jun., Jul., Aug., Sep.). Storm flow analyses incorporated all possible sampled storm events (prerestoration: *n* = 4 (North Up) or *n* = 5 (North Down, all Middle Macatawa sites); postrestoration: *n* = 6 (all Haworth and Middle Macatawa sites)).

Normality was tested using the Shapiro–Wilk test and equal variance was tested using the Brown–Forsythe test. Data not meeting test assumptions of normality and equal variance were transformed prior to analysis. Statistical significance was indicated by *p*-values < 0.05. Trends of marginal significance were indicated by *p*-values < 0.10. All statistical tests were performed using SigmaPlot 13.0 (Systat Software, Inc.: San Jose, CA, USA).

### 2.4. Lake Macatawa

Water quality monitoring in the lake was conducted at five sites during spring, summer, and fall 2017 (Figure 1D). At each sampling location, general water quality measurements (DO, temperature, pH, specific conductivity, TDS, ORP, and turbidity) were taken using a YSI 6600 sonde at the surface, middle, and near bottom of the water column. Water transparency was measured as Secchi disk depth. Water samples were collected from the surface and near-bottom of the water column using a Van Dorn Bottle and analyzed for SRP, TP, and chlorophyll *a* (Chl *a*). Additional Lake Macatawa water samples for ${\mathrm{NO}}_{3}^{-}$, NH_3_, and TKN were collected for the first time in 2017. Samples also were taken for phytoplankton community composition and archived for possible future analysis.

Water for SRP and ${\mathrm{NO}}_{3}^{-}$ analysis was syringe-filtered through acid washed 0.45-μm membrane filters into scintillation vials and refrigerated until analysis. SRP was refrigerated and ${\mathrm{NO}}_{3}^{-}$ frozen until analysis. NH_3_ and TKN were acidified with sulfuric acid and kept at 20 °C until analysis. SRP, TP, ${\mathrm{NO}}_{3}^{-}$, NH_3_, and TKN were analyzed as previously described. Chl *a* samples were filtered through GFF filters and frozen until analysis on a Shimadzu UV-1601 spectrophotometer (Shimadzu Corp.: Kyoto, Japan) [12].

## 3. Results

### 3.1. Wetland Restoration: Middle Macatawa Property

#### 3.1.1. 2017-Only Data

Mean baseflow (and storm flow concentrations) of DO were generally good, averaging ~9.5 to 10.5 mg/L (Table 2). Mean specific conductivity was high: >600 µS/cm at all sites during baseflow but declined by half, along with TDS, during storm events (Table 2). Mean turbidity concentrations were 9–15 NTU during baseflow but increased by ~50-fold during storms (Table 2), presumably because of increased erosion from both fields and stream banks.

**Figure 2:**
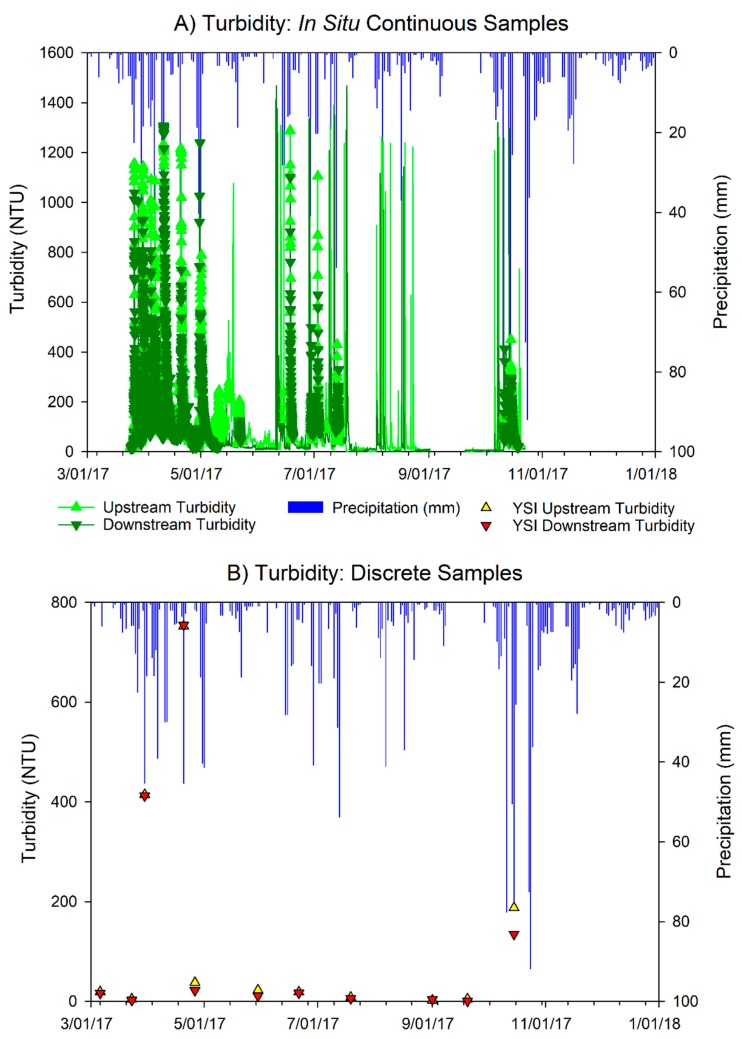
Daily precipitation and turbidity (NTU) during 2017 sampling season at the Middle Macatawa Upstream and Downstream sites. (**A**) Turbidity data collected every half hour and (**B**) discrete baseflow and storm turbidity measurements were taken during monthly baseflow sampling. Hourly precipitation data (panels A and B) were retrieved from the National Climatic Data Center website and summed by day.

We measured turbidity with both discrete grab samples during storm events (*n* = 3), as well as with in situ turbidity sensors. The in situ meters detected higher turbidity events that were not captured during monthly sampling during the mid-June to mid-July period and the late October precipitation events (Figure 2). The turbidity peaks align well with storm events, as evidenced by 2017 precipitation data collected from the National Climatic Data Center (NCDC) website for Tulip City Airport (Figure 2). In situ turbidity meter data gaps in early fall are due to low water levels near the sonde.

**Figure 3:**
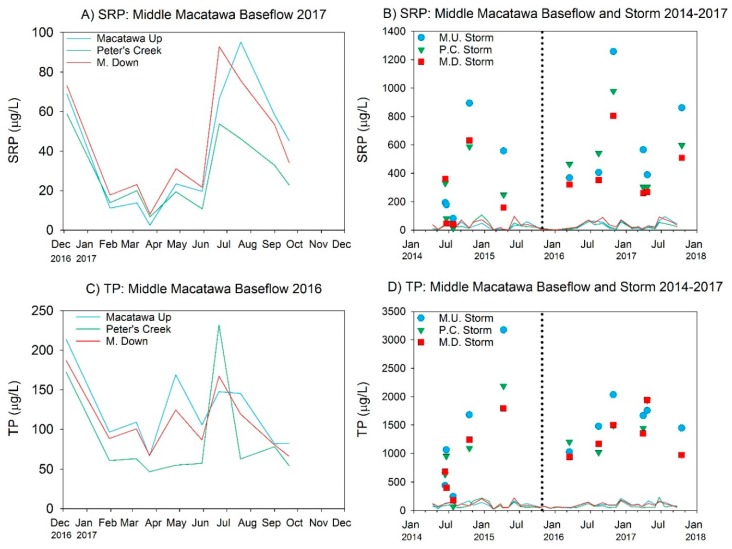
SRP and TP concentrations measured at Middle Macatawa restoration site in 2017 (**A**,**C**) and over total project history (**B**,**D**). Colored data lines in (**A,C**) magnify the 2017 baseflow data shown in (**B,D**). Symbols represent storm events. Vertical dotted lines represent approximate completion date of wetland restoration construction.

**Figure 4:**
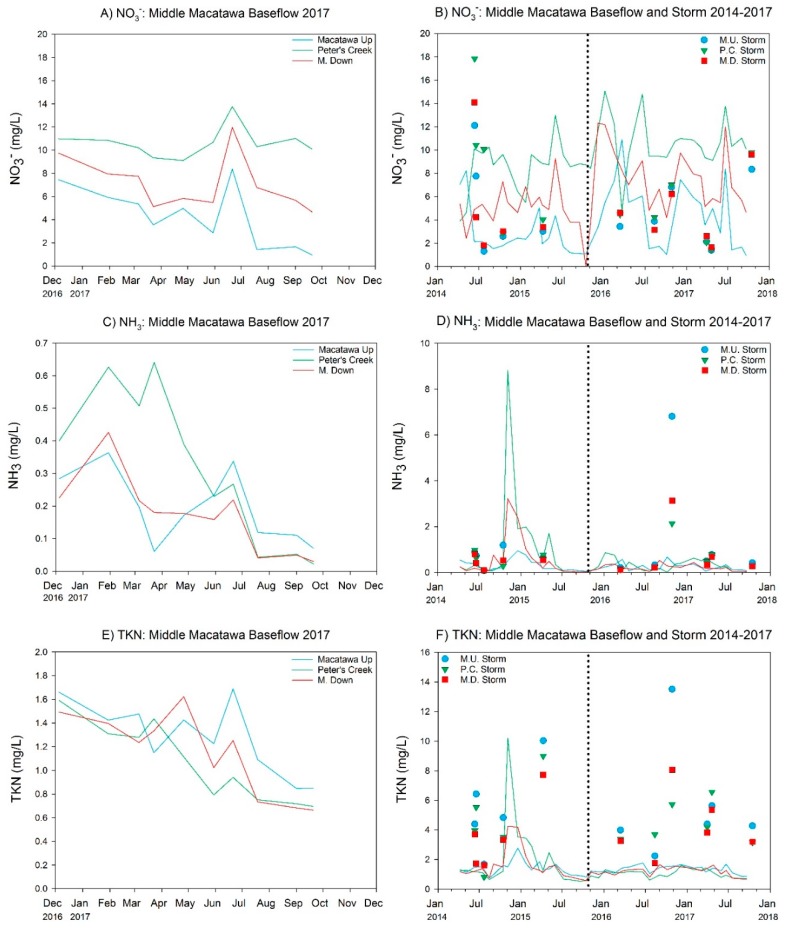
Nitrate (${\mathrm{NO}}_{3}^{-}$), ammonia (NH_3_), and total Kjeldahl nitrogen (TKN) concentrations measured at the Middle Macatawa restoration site in 2017 (**A**,**C**,**E**) and over total project history (**B**,**D**,E). See Figure 3 caption for more explanation.

Nutrient concentrations were relatively high during baseflow at all three sites, with mean SRP concentrations between 29 and 43 µg/L, and mean TP ranging between 88 and 122 µg/L (Table 3, Figure 3A,C), indicative of highly eutrophic conditions. Mean nitrate concentrations were also very high during baseflow (Table 3, Figure 4A); baseflow concentrations of ammonia and TKN were much lower than nitrate (Table 3, Figure 4C,E), but still potentially problematic. Nutrient concentrations changed considerably with storm runoff, with SRP, TP, ammonia, and TKN increasing substantially relative to baseflow but nitrate declining (although still high) (Table 3 and Figure 3B,D and Figure 4B,D,F). Storm events results in mean SRP concentrations ranging from 347 to 605 µg/L while mean TP concentrations were well above 1000 µg/L (Table 3, Figure 3D), which is 20× the interim TMDL target.

**Figure 5:**
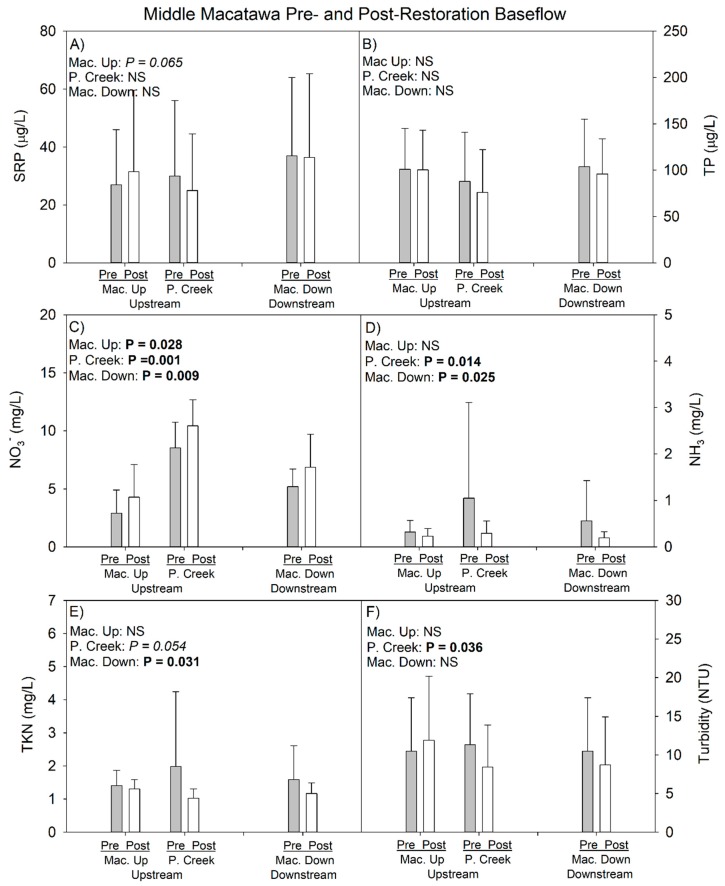
Mean (1 SD) Middle Macatawa pre- and postrestoration nutrient value comparison at baseflow in 2017 sampling year. *p*-values in top left corner of each panel represent pre- vs. postrestoration statistical analysis within each site. Mac. Up = Macatawa Upstream site; P. Creek = Peters Creek; Mac. Down = Macatawa Downstream site. See Figure 1B for site locations.

**Figure 6:**
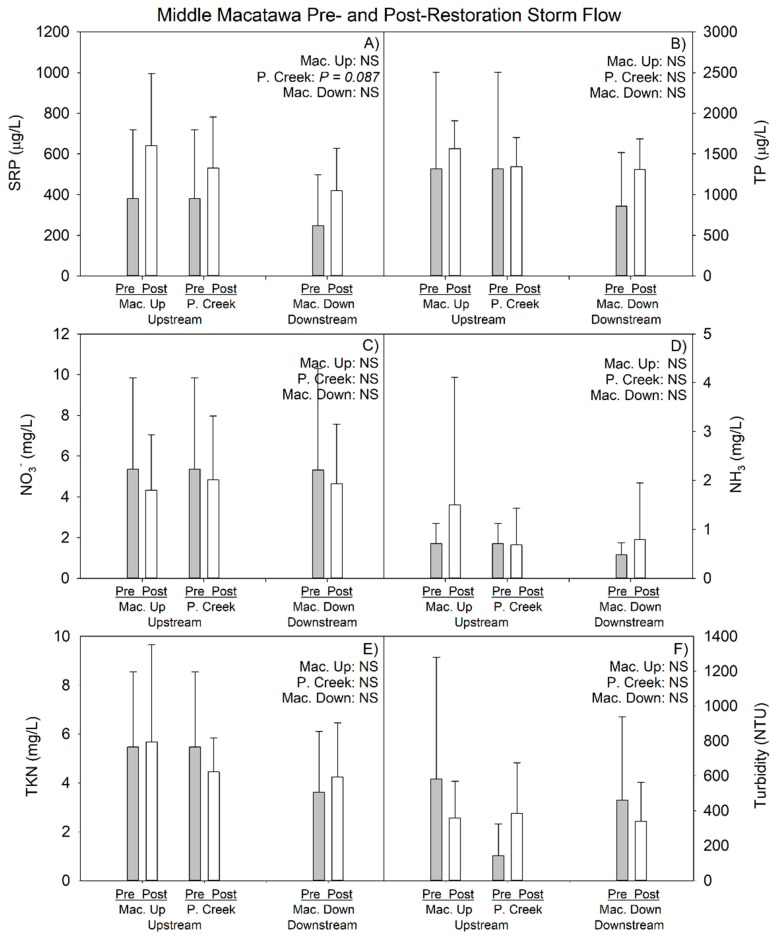
Mean (1 SD) Middle Macatawa pre- and postrestoration nutrient comparison at storm flow as of 2017 sampling year. *p*-values in top left corner of each panel represent pre- vs. postrestoration statistical analysis within each site. Mac. Up = Macatawa Upstream site; P. Creek = Peters Creek; Mac. Down = Macatawa Downstream site. See Figure 1B for site locations.

There was no indication that the downstream site had better water quality than either of the upstream sites at baseflow (Figure 5); indeed, the only statistically significant contrast for P among the three sampling locations showed differences in TP among the two upstream sites (Mac Up > Peters Creek; *p* = 0.031; Kruskal–Wallis; Figure 5B). The only other statistically significant difference involved nitrate, which was greater in Peters Creek than either Macatawa Up or Down, and Macatawa Down was greater than Macatawa Up (*p* < 0.001; ANOVA). There were no statistically significant differences among the three sampling locations during stormflow (*p* > 0.05; Figure 6).

#### 3.1.2. 2014–2017 Data (Pre- vs. Postrestoration Comparison)

Mean water quality values during baseflow, based on the prerestoration and postrestoration time periods, reveals generally similar patterns and values at all three sites, and values generally similar to the 2017 data for DO, specific conductivity, and TDS (Table 2 and Table 4). Pre- and postrestoration mean SRP and TP concentrations were relatively similar at all three sites, whereas mean nitrate concentrations increased postrestoration and both ammonia and TKN concentrations declined following restoration (Table 5, Figure 4) during baseflow.

Water quality trends were more complex when comparing pre- vs. postrestoration periods during storm events. All three sites had lower temperature (possibly due to differences in storm event timing, as three of the five prerestoration storms were in the summer, whereas half of the postrestoration storms were in early to mid-spring) and higher DO in the postrestoration period (Table 4). In addition, specific conductivity and TDS declined following restoration at all three sites during storm flow; turbidity, on the other hand, was higher at the Peters Creek site during the postrestoration period, but lower at the other two sites following restoration (Table 4). Indeed, Peters Creek appears to behave like an outlier in the postrestoration period, showing declines in mean ammonia and TKN, while the other two sites had increases during storm flow (Table 5). All three sampling locations showed higher mean SRP and TP concentrations following restoration, although mean nitrate values did decline in the postrestoration period (Table 5).

We used a subset of our overall data set to determine if the differences in water quality between the pre- vs. postrestoration periods were statistically significant (Table 6). We selected 16 baseflow sampling dates and three to five stormflow sampling dates that corresponded in sampling date between the pre- and postrestoration monitoring periods at each site, and compared differences using inferential statistics. The results show few statistically significant differences, which is not surprising given that the wetland restoration was only recently completed and there was very high variance in the data.

For baseflow periods, SRP was marginally greater (*p* < 0.10) in the postrestoration period at the Macatawa Up site, but was not different at the other two sites (Table 6). Nitrate showed the most consistent and statistically significant pattern, being greater postrestoration at all three sites. Ammonia and TKN were significantly greater in pre- vs. postrestoration periods at Macatawa Down and mixed at Peters Creek (Table 6). Turbidity declined following restoration at Peters Creek (Table 6) but there was no significant difference at the other two sites. For storm event periods, there was only one significant difference, with SRP marginally greater following restoration at the Peters Creek site (Table 6).

Comparison of just the downstream site to the two upstream sites for the postrestoration period indicated slightly lower or equivalent mean nutrient and turbidity concentrations at all sites (Table 6), suggesting no obvious impact of restoration on water quality to date (Figure 5 and Figure 6).

### 3.2. Wetland Restoration: Haworth Property

#### 3.2.1. 2017-Only Data

Baseflow water quality parameters measured in the North Branch at the Haworth site were generally similar to baseflow observations at the Middle Macatawa property, although absolute turbidity values were approximately half at Haworth (Table 7). Similar to the Middle Macatawa site, storm events diluted specific conductivity and TDS values but increased turbidity (Table 7).

**Figure 7:**
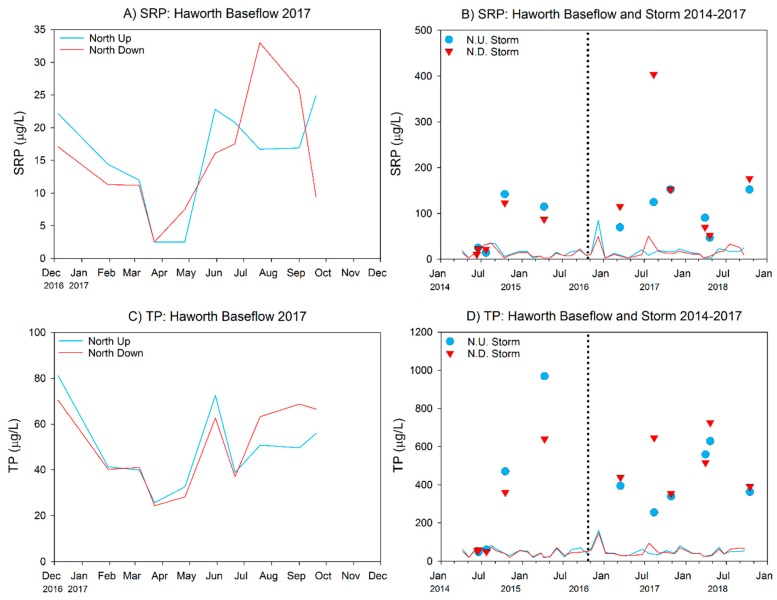
SRP and TP concentrations measured at Haworth wetland for 2017 (**A**,**C**) and total project history (**B**,**D**). Colored data lines in (A,C) magnify the 2017 baseflow data shown in (B,D). Symbols represent storm events. Vertical dotted lines represent approximate completion date of wetland restoration construction.

**Figure 8:**
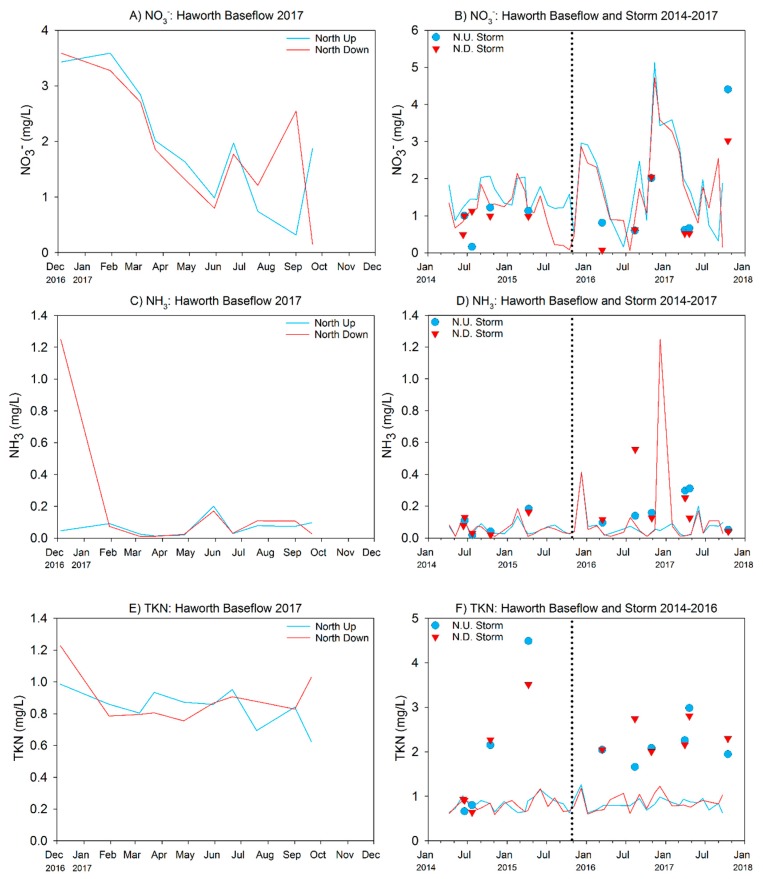
Nitrate (NO_3_^−^), ammonia (NH_3_), and total Kjeldahl nitrogen (TKN) concentrations measured at the Haworth wetland for 2017 (**A**,**C**,**E**) and total project history (**B**,**D**,E). See Figure 7 caption for more explanation.

Nutrient concentrations at baseflow were considerably lower than those observed at the Middle Macatawa property (Table 8 vs. Table 4), although absolute concentrations of TP and nitrate were still relatively high, and indicative of eutrophic conditions (Table 8, Figure 7 and Figure 8). There were no statistically significant differences between up- and downstream sites for any of the nutrients (*p* > 0.05). SRP and TP increased ~10× and TKN increased ~3× under storm conditions (Table 8). Similar to baseflow conditions, there were no statistically significant differences in the upstream–downstream comparisons for any of the water quality parameters during our measured storm events (Table 8), suggesting that the effect of runoff is overwhelming any localized impact of restoration to date.

#### 3.2.2. 2014–2017 Data (Pre- vs. Postrestoration Comparison)

At baseflow, temperature and DO were slightly lower post- compared to prerestoration; however, these differences also were reflected at the downstream site (Table 9), so they are likely due to annual differences in climate. All nutrient concentrations at baseflow, with the exception of nitrate, were very similar in the pre- and postrestoration periods (Table 10). Nitrate was elevated in the postrestoration period, but again this change was reflected at both the upstream and downstream locations, suggesting restoration activity was not responsible for the difference.

**Figure 9:**
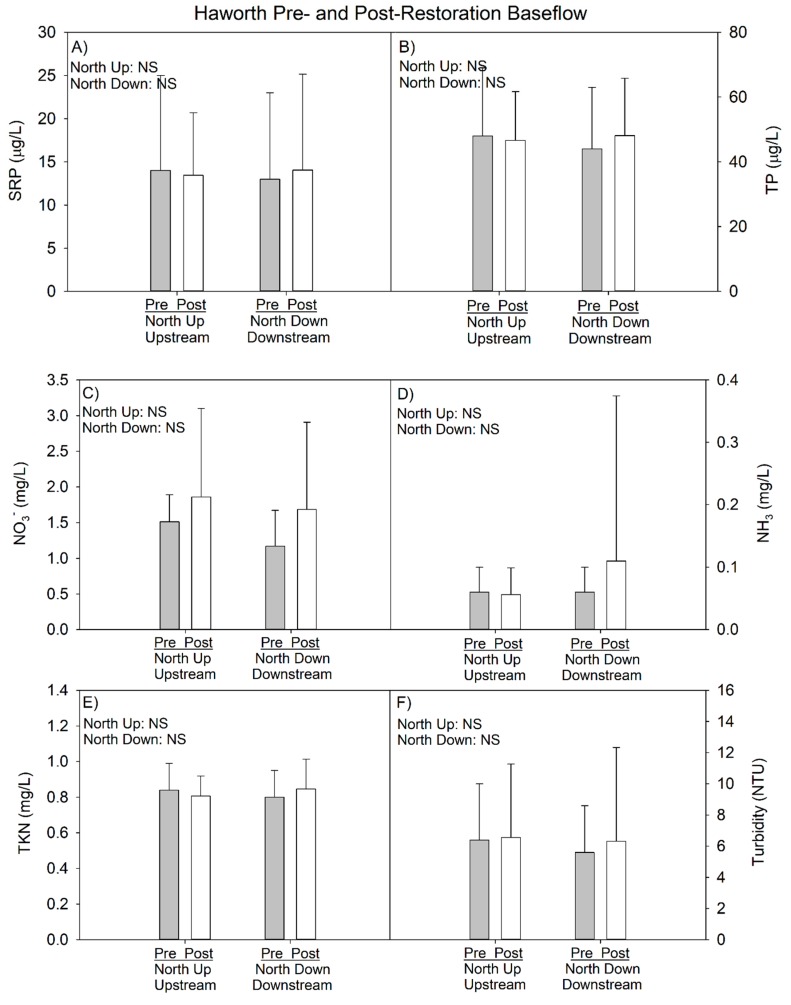
Haworth pre- and postrestoration water chemistry comparison at baseflow as of 2017 sampling year. Error bars represent 1 SD. *p*-values in top left corner of each panel represent pre- vs. post-restoration statistical analysis within each site. See Figure 1C for site locations.

**Figure 10:**
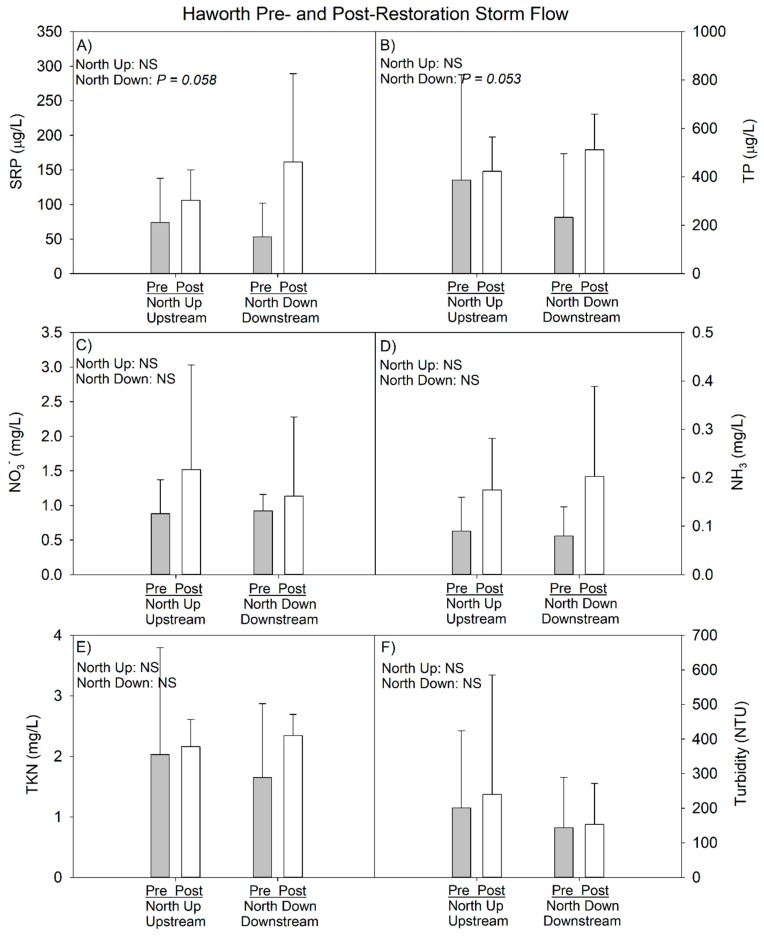
Haworth pre- and postrestoration water chemistry comparison at storm flow as of 2017 sampling year. Error bars represent 1 SD. *p*-values in top left corner of each panel represent pre- vs. postrestoration statistical analysis within each site. See Figure 1C for site locations.

During storm events, we observed deteriorated water quality in the post- vs. prerestoration periods, as mean turbidity and nutrient concentrations increased following restoration, regardless of whether the comparison was within the upstream or downstream sites; however, none of the increases at the upstream site were statistically significant (Table 11). At the downstream site, the nutrient concentrations were again all greater post- vs. prerestoration, but in this case both SRP and TP were marginally significant (*p* < 0.10). Comparison of just the downstream site to the upstream site for the postrestoration period indicated no differences at baseflow but higher P and nitrate concentrations were measured at the downstream site at storm flow (Figure 9 and Figure 10).

### 3.3. Lake Macatawa

**Figure 11:**
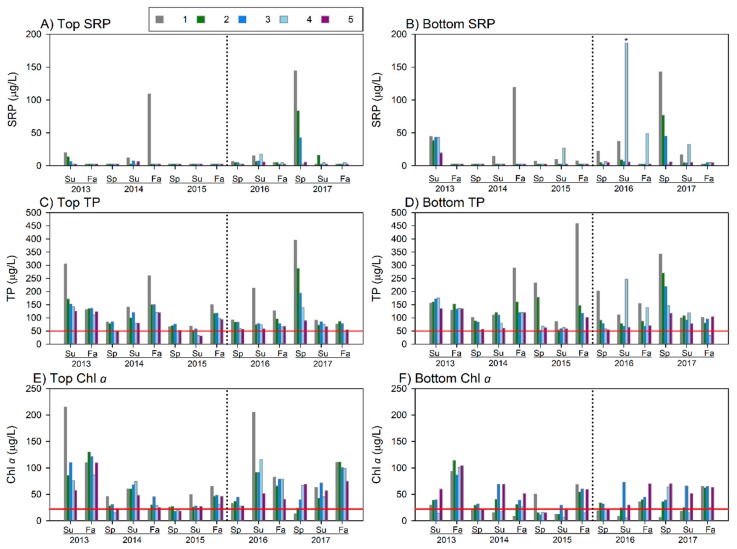
SRP, TP, and Chl *a* concentrations measured at the five monitoring stations in Lake Macatawa from 2013 through 2017. The red horizontal lines on TP indicate the interim TMDL goal of 50 μg/L; red horizontal lines on Chl *a* figures indicate the hypereutrophic boundary of 22 μg/L used by MDEQ for assessing chlorophyll in Lake Macatawa (Holden 2014). Summer 2016 site 4 bottom depth SRP sample (**B**, asterisked) is a likely outlier due to sediment disturbance.

In 2017, surface and bottom SRP and TP concentrations were much higher in spring than in summer or fall (Figure 11A–D), possibly due to input from watershed runoff, as well as less abundant phytoplankton in spring to take up the nutrients from the water column. Mean surface Chl *a* concentrations in 2017 peaked in fall, in contrast to 2016 when they peaked in summer (Figure 11). Surface and bottom levels of SRP, TP, and Chl *a* have not declined postrestoration, and both TP and Chl *a* continue to exceed eutrophic thresholds (Figure 11).

## 4. Discussion

Nonpoint source pollution, especially from agricultural areas, is a major factor causing eutrophication and associated algal blooms in regions throughout the world [13,14,15]. Despite a wide variety of mitigation options [15] and planning tools [16], impaired water quality continues to plague water bodies on a global basis. The proliferation of cyanobacterial blooms, and their associated toxins, is anticipated to worsen when combined with climate change [17].

Many lakes and rivers in the upper Midwest, including portions of the Laurentian Great Lakes, have experienced significant eutrophication over the past few decades. Excessive nutrient inputs, exacerbated by extreme precipitation events that mobilize recently applied, and readily bioavailable, fertilizer [4,18,19], have contributed to potentially harmful algal blooms, hypoxic and anoxic conditions, and associated ecological and socioeconomic impairments. These challenges persist despite the investment of hundreds of millions of dollars in BMPs, begging several questions: Are we using ineffective BMPs? Are we locating BMPs in the wrong areas? Should we be more patient for the BMPs to become more effective? Does the intensity of agricultural land use overwhelm the assimilative capacity of the BMPs? Or perhaps, as implied by Kleinman et al. [16], is there sufficient satisfaction with implementation of the management practice (an output) instead of its effectiveness (an outcome) that we do not push harder for better outcomes?

Lake Macatawa and its watershed is one of the most impaired systems in Michigan. To address excess sediment and P loading, a public–private partnership was formed. The project partners have implemented a number of BMPs including wetland creation, bank stabilization, two-stage ditches, tile drain control structures, and no-till rotations. Monitoring has been incorporated to assess the performance of BMPs, with the explicit recognition that the problems facing the watershed accumulated over 150 years, and they would not be resolved overnight. It is clear from our data that in the few years since BMPs have been implemented, there has been little improvement in water quality either downstream of the created wetlands or in the downstream receiving water body (Lake Macatawa). This is not surprising given the short time period since implementation; continued monitoring will reveal if water quality improves over time as BMPs both increase in number across the watershed and mature, resulting in greater effectiveness.

The data from tributary monitoring to date indicate that in terms of DO, stream conditions are passable, as DO concentrations <5 mg/L, indicative of stress to many fishes, were not observed in our sampling. However, conductivity levels above 600 µS/cm were common, and they are generally indicative of human-induced stress in aquatic ecosystems [20]. In addition, phosphorus concentrations were extremely high, especially during storm events, and nitrate concentrations in excess of 10 mg/L, which can be toxic to warm-blooded animals under certain conditions, were measured in Peters Creek. These high nitrate levels are likely associated with the application of fertilizer on fields [21,22].

We identify several caveats in our results, which help place our findings in a broader context. First, because BMP construction occurred less than two years prior to our monitoring, it is possible that the short-term impacts of soil movement during restoration activities resulted in increased nutrients. If so, these concentrations should decline once the restored wetlands become mature and are fully functional. This point applies to other BMPs, such as two-stage ditches [23] and bank stabilization efforts.

The TMDL for Lake Macatawa was established in 1999, before the impacts of climate change were widely recognized. Today, the concerns over episodic storm events, and their ability to move sediment and nutrients, are much more acute [18,24,25]. Hence, the 50 µg/L TP target may be even harder to obtain than previously realized, and effectiveness of BMPs becomes ever more important. The mean surface TP in Lake Macatawa ranged from 78 to 221 µg/L (Table 11), far above the target. Concentrations do decline at sites farther westward in the lake, presumably due to the settling out of particles and dilution from high quality Lake Michigan water advecting into the western end of Lake Macatawa. Despite occasional spikes in bottom water SRP and TP, overall there was no evidence of systemic internal P loading in Lake Macatawa, which if present, would be indicated by very high concentrations of SRP and/or TP (>400 µg/L) in bottom waters, as we have measured in other west Michigan drowned-river mouth lakes [26,27]. The influence of groundwater on the lake’s ecology has not been addressed and was not considered a major factor as part of the lake’s TMDL [10], presumably because the septic systems in direct lake discharge sub-basin (Figure 1) have been replaced by sanitary sewers.

Both surface and bottom Chl *a* values frequently exceeded the 22 μg/L hypereutrophic threshold commonly used by MDEQ in its assessments of Lake Macatawa [9]. Mean Secchi disk depths indicated low transparency throughout the year, less than 1 m, suggesting eutrophic to hypereutrophic conditions [28]; high sediment loads may help keep algal blooms from becoming even more problematic due to reduced light transmission [29]. The lack of improvement in lake condition is not surprising as it often takes years, if not decades, for lake conditions to improve once the stressors are removed, and in many cases, the stressors remain in place but at reduced levels, exacerbating lake impairment [5,30].

The lack of improvement in the watershed and lake in 2017 can be attributed to at least four reasons: (1) Restoration is still very recent, and until the restored sites are fully functional, which should take a number of years, it is unreasonable to expect a demonstrable change; (2) the two created wetland restoration sites have relatively small footprints and volume holding capacity compared to the entire watershed; the two sites have a combined area of 0.45 km^2^ compared to the watershed area of 464 km^2^. Given the volume of water moving through the Macatawa River, especially during storm events, the ability to detect a signal from the noise may be very difficult at any one particular site; (3) the natural environment is variable, so it will take a number of years to detect a robust trend at any site, regardless of direction; and (4) 2017 was a dry year (43% lower than long-term average), thereby resulting in fewer opportunities for the wetlands to serve as filtering and retention basins to remove transport of pollutants.

Future needs in this watershed include continued monitoring of water quality conditions and implementation of targeted agricultural BMPs. For example, tile drain effluent appears to be an additional source of P (and maybe N) [31], but has not received adequate attention in the past and is now being recognized as a factor contributing to toxic algal blooms in the western basin of Lake Erie [32,33]. We are in the process of developing a SWAT model for the watershed (Iavorivska, in preparation) which will allow managers to test scenarios to assess potential efficacy of different BMPs placed in different locations.

## 5. Conclusions

In an effort to restore the ecological health of phosphorus- and sediment-impaired Lake Macatawa, two wetland restoration projects were constructed. This study assesses their effectiveness at improving water quality two years following construction.

(1) Phosphorus and sediment concentrations increased dramatically in the watershed following storm events, suggesting BMPs may play a critical role in improving water quality in this watershed.
(2) However, in both constructed wetlands, there was no evidence of water quality improvement to date when comparing pre- to postconstruction data. In addition, Lake Macatawa, the receiving water body in this watershed, also showed no improvement in water quality.
(3) Possible reasons why water quality has not improved: (a) two years is an insufficient amount of time to detect meaningful ecological changes; (b) wetland footprints are too small to make a difference in water quality given the loads currently being delivered; (c) relatively dry years following construction have limited the ability of the wetlands to have their greatest impact; and d) high natural variability may mask the ability to detect change over a short period of record.

Additional BMPs, such as two-stage ditches, winter cover crops, and tile drain management are being implemented throughout the watershed to complement the constructed wetlands. Continued monitoring will allow us to determine the effectiveness of these BMPs in reducing nonpoint source pollution, and whether those reductions are resulting in improved lake water quality.

## Figures and Tables

**Table 1:** Precipitation summary for storm events sampled in 2017.

	30 March 2017	20 April 217	15 October 2017
Rainfall (cm)	6.17	8.15	16.38
Duration (h)	20	21	51
Intensity (cm/h)	0.31	0.39	0.32

**Table 2:** Mean (1 Standard Deviation (SD)) values of water quality parameters at the Middle Macatawa wetland restoration site during the 2017 postrestoration sampling year (Dec. 2016–Nov. 2017). Storm flow data shown in gray shade.

Flow	Site	*n*	Temp. (C)	DO (mg/L)	SpCond (µS/cm)	TDS (g/L)	Turbidity (NTU)
Base	Mac. Up	10	13.65 (8.70)	9.42 (2.31)	686 (111)	0.446 (0.072)	15.3 (10.9)
	Peters Creek	10	12.84 (7.37)	10.32 (2.01)	637 (59)	0.414 (0.038)	9.1 (5.6)
	Mac. Down	10	12.54 (8.17)	10.21 (2.50)	704 (89)	0.457 (0.058)	10.9 (7.9)
Storm	Mac. Up	3	11.16 (5.56)	9.91 (2.03)	328 (99)	0.213 (0.065)	451.5 (284.0)
	Peters Creek	3	10.54 (4.34)	10.41 (1.65)	311 (148)	0.202 (0.097)	505.9 (406.2)
	Mac. Down	3	11.08 (5.64)	9.94 (2.22)	334 (114)	0.217 (0.074)	433.9 (310.6)

**Table 3:** Mean (1 SD) values for phosphorus (TP and SRP) and nitrogen (nitrate (NO_3_^−^), ammonia (NH_3_), and total Kjeldahl nitrogen (TKN)) at the Middle Macatawa wetland restoration site from Dec. 2016–Nov. 2017. Storm flow data shown in gray shade.

Flow	Site	*n*	SRP (µg/L)	TP (µg/L)	${NO}_{3}^{-}$ (mg/L)	NH_3_ (mg/L)	TKN (mg/L)
Base	Mac. Up	10	40 (31)	122 (46)	4.26 (2.58)	0.19 (0.11)	1.28 (0.30)
	Peters Creek	10	29 (18)	88 (62)	10.64 (1.28)	0.32 (0.23)	1.06 (0.33)
	Mac. Down	10	43 (29)	109 (41)	7.10 (2.31)	0.17 (0.12)	1.14 (0.35)
Storm	Mac. Up	3	605 (239)	1622 (159)	3.95 (3.81)	0.56 (0.19)	4.76 (0.75)
	Peters Creek	3	402 (169)	1455 (477)	4.44 (4.66)	0.53 (0.25)	4.63 (1.75)
	Mac. Down	3	347 (141)	1421 (488)	4.62 (4.36)	0.43 (0.22)	4.13 (1.11)

**Table 4:** Grand means (1 SD) of water quality parameters at the Middle Macatawa wetland restoration site. Values in the top half of each cell represent prerestoration (Apr. 2014–Sep. 2015); values in the bottom half represent postrestoration (Oct. 2015–Nov. 2017). Storm flow data shown in gray shade.

Flow	Site	Period	*n*	Temp. (C)	DO (mg/L)	SpCond (µS/cm)	TDS (g/L)	Turbidity (NTU)
Base	Mac. Up	Pre	18	12.17 (7.40)	10.53 (2.39)	765 (240)	0.497 (0.156)	10.5 (6.9)
		Post	22	12.97 (8.14)	9.78 (2.51)	757 (120)	0.492 (0.078)	11.9 (8.3)
	Peters Creek	Pre	18	12.35 (7.38)	10.45 (2.39)	665 (163)	0.432 (0.106)	11.3 (6.6)
		Post	22	12.26 (7.12)	10.38 (2.15)	667 (79)	0.433 (0.051)	8.4 (5.5)
	Mac. Down	Pre	18	12.17 (7.40)	10.53 (2.39)	765 (240)	0.497 (0.156)	10.5 (6.9)
		Post	22	12.00 (7.62)	10.34 (2.43)	727 (83)	0.472 (0.054)	8.7 (6.2)
Storm	Mac. Up	Pre	3	14.26 (6.78)	7.43 (2.68)	444 (207)	0.288 (0.135)	581.7 (697.8)
		Post	6	11.99 (7.05)	9.49 (2.38)	392 (124)	0.255 (0.081)	357.9 (210.6)
	Peter’s Creek	Pre	2	17.00 (3.75)	7.49 (0.81)	460 (201)	0.299 (0.130)	141.6 (182.5)
		Post	6	11.71 (6.53)	9.92 (2.50)	327 (128)	0.213 (0.084)	384.9 (290.7)
	Mac. Down	Pre	3	14.00 (6.66)	7.88 (2.42)	481 (201)	0.313 (0.130)	462.2 (475.9)
		Post	6	11.95 (6.91)	9.64 (2.34)	364 (130)	0.236 (0.085)	338.8 (223.9)

**Table 5:** Grand means (1 SD) of nutrient concentrations at the Middle Macatawa wetland restoration site. Values in the top half of each cell represent prerestoration (Apr. 2014–Sep. 2015) and values in the bottom half represent post-restoration (Oct. 2015–Nov. 2017). Storm flow data shown in gray shade.

Flow	Site	Period	*n*	SRP (µg/L)	TP (µg/L)	NO_3_^−^ (mg/L)	NH_3_ (mg/L)	TKN (mg/L)
Base	Mac. Up	Pre	18	27 (19)	101 (44)	2.90 (2.00)	0.32 (0.25)	1.41 (0.46)
		Post	22	32 (28)	100 (43)	4.29 (2.83)	0.23 (0.17)	1.31 (0.28)
	Peter’s Creek	Pre	18	30 (26)	88 (53)	8.54 (2.19)	1.05 (2.06)	1.98 (2.26)
		Post	22	25 (20)	76 (46)	10.43 (2.25)	0.29 (0.27)	1.03 (0.28)
	Mac. Down	Pre	18	37 (27)	104 (51)	5.20 (1.51)	0.56 (0.87)	1.59 (1.02)
		Post	22	36 (29)	96 (38)	6.86 (2.83)	0.19 (0.14)	1.17 (0.32)
Storm	Mac. Up	Pre	5	381 (339)	1320 (1181)	5.35 (4.49)	0.71 (0.41)	5.47 (3.07)
		Post	6	641 (355)	1567 (340)	4.32 (2.71)	1.50 (2.61)	5.67 (3.99)
	Peters Creek	Pre	5	381 (339)	1320 (1181)	5.35 (4.49)	0.71 (0.41)	5.47 (3.07)
		Post	6	532 (250)	1346 (358)	4.83 (3.13)	0.69 (0.75)	4.45 (1.38)
	Mac. Down	Pre	5	248 (251)	860 (657)	5.31 (4.99)	0.48 (0.25)	3.62 (2.48)
		Post	6	420 (209)	1313 (376)	4.64 (2.92)	0.79 (1.16)	4.24 (2.20)

**Table 6:** Pre- vs. postrestoration statistical analyses of water quality at Middle Macatawa sites at baseflow and storm flow. To remove potential bias of pre- vs. postrestoration samples collected from different time periods, baseflow tests incorporated an equal number of samples from identical months in multiyear pre- and postrestoration periods (Apr., Jun., Jul., Sep., Oct., Nov., Dec., Jan., Feb., Mar., Apr., May, Jun., Jul., Aug., Sep.). Storm flow tests incorporated all possible sampled storm events.

		Mac. Up			Peter’s Creek			Mac. Down		
Flow	Parameter	Transform	*p*-Value	Notes	Transform	*p*-Value	Notes	Transform	*p*-Value	Notes
Base	SRP	-	*0.065*	*post > pre*	sqrt	0.925	NS	-	0.262	NS
	TP	-	0.572	NS	-	0.460	NS	-	0.979	NS
	NO_3_^−^	-	**0.028**	**post > pre**	**x^2^**	**0.001**	**post > pre**	**-**	**0.009**	**post > pre**
	NH_3_	-	0.120	NS	**log**	**0.014**	**pre > post**	**log**	**0.025**	**pre > post**
	TKN	-	0.286	NS	*1/x*	*0.054*	*post > pre*	**log**	**0.031**	**pre > post**
	Turbidity	-	0.495	NS	-	**0.036**	**pre > post**	-	0.411	NS
Storm	SRP	sqrt	0.178	NS	-	*0.087*	*post > pre*	sqrt	0.155	NS
	TP	sqrt	0.537	NS	-	0.336	NS	-	0.184	NS
	NO_3_^−^	-	0.650	NS	-	0.169	NS	sqrt	0.873	NS
	NH_3_	log	0.701	NS	log	0.867	NS	log	0.943	NS
	TKN	sqrt	0.953	NS	-	0.938	NS	-	0.668	NS
	Turbidity	-	0.469	NS	-	0.321	NS	-	0.599	NS

Statistically significant differences shown in bold.

**Table 7:** Mean (1 SD) values of water quality parameters at the Haworth wetland restoration site for the 2017 sampling year. Storm flow data shown in gray shade.

Flow	Site	*n*	Temp. (C)	DO (mg/L)	SpCond (µS/cm)	TDS (g/L)	Turbidity (NTU)
Base	North Up	10	12.10 (7.84)	9.27 (2.77)	767 (259)	0.498 (0.169)	6.8 (5.0)
	North Down	10	12.16 (7.53)	9.18 (2.83)	809 (192)	0.526 (0.125)	4.9 (3.5)
Storm	North Up	3	11.15 (5.31)	9.59 (2.60)	307 (85)	0.199 (0.055)	390.8 (478.5)
	North Down	3	11.62 (5.51)	9.23 (2.54)	332 (46)	0.215 (0.030)	204.8 (161.4)

**Table 8:** Mean (1 SD) values of nutrient concentrations at the Haworth restoration site for the 2017 sampling year. Storm flow data shown in gray shade.

Flow	Site	*n*	SRP (µg/L)	TP (µg/L)	NO_3_^−^ (mg/L)	NH_3_ (mg/L)	TKN (mg/L)
Base	North Up	10	16 (8)	49 (17)	1.94 (1.10)	0.07 (0.06)	0.84 (0.11)
	North Down	10	15 (9)	50 (18)	1.92 (1.10)	0.18 (0.38)	0.89 (0.14)
Storm	North Up	3	97 (53)	517 (138)	1.90 (2.17)	0.22 (0.15)	2.40 (0.53)
	North Down	3	100 (67)	544 (169)	1.36 (1.45)	0.14 (0.11)	2.42 (0.34)

**Table 9:** Grand mean (1 SD) values of water quality parameters at the Haworth wetland restoration site in pre- and postrestoration sampling periods. Values in the top half of each cell represent prerestoration (Apr. 2014–Sep. 2015); values in the bottom half represent postrestoration (Oct. 2015–Nov. 2017). Storm flow data shown in gray shade.

Flow	Site	Period	*n*	Temp. (C)	DO (mg/L)	SpCond (µS/cm)	TDS (g/L)	Turbidity (NTU)
Base	North Up	Pre	18	12.38 (7.11)	11.02 (3.89)	843 (144)	0.548 (0.093)	6.4 (3.6)
		Post	22	11.48 (7.67)	9.70 (2.78)	801 (200)	0.521 (0.130)	6.5 (4.7)
	North Down	Pre	18	11.93 (6.96)	10.32 (3.36)	844 (194)	0.549 (0.126)	5.6 (3.0)
		Post	22	11.54 (7.72)	9.52 (2.76)	825 (148)	0.537 (0.096)	6.3 (6.0)
Storm	North Up	Pre	3	13.80 (5.92)	7.77 (2.29)	432 (283)	0.281 (0.184)	200.7 (223.6)
		Post	6	12.15 (6.95)	9.21 (2.63)	389 (107)	0.253 (0.069)	240.0 (345.3)
	North Down	Pre	3	13.80 (6.06)	7.84 (2.32)	478 (150)	0.310 (0.098)	143.6 (146.0)
		Post	6	12.44 (7.05)	8.97 (2.83)	415 (98)	0.270 (0.064)	153.4 (118.2)

**Table 10:** Grand mean (1 SD) values of selected nutrient concentrations at the Haworth restoration site in pre- and postrestoration sampling periods. Values in the top half of each cell represent prerestoration (Apr. 2014–Sep. 2015); values in the bottom half represent postrestoration (Oct. 2015–Nov. 2017). Storm flow data shown in gray shade.

Flow	Site	Period	*n*	SRP (µg/L)	TP (µg/L)	NO_3_^−^ (mg/L)	NH_3_ (mg/L)	TKN (mg/L)
Base	North Up	Pre	18	14 (11)	48 (21)	1.51 (0.38)	0.06 (0.04)	0.84 (0.15)
		Post	22	13 (7)	47 (15)	1.86 (1.24)	0.06 (0.04)	0.81 (0.11)
	North Down	Pre	18	13 (10)	44 (19)	1.17 (0.50)	0.06 (0.04)	0.80 (0.15)
		Post	22	14 (11)	48 (18)	1.68 (1.23)	0.11 (0.26)	0.85 (0.17)
Storm	North Up	Pre	4	74 (64)	387 (435)	0.88 (0.49)	0.09 (0.07)	2.03 (1.77)
		Post	6	106 (44)	423 (142)	1.52 (1.52)	0.17 (0.11)	2.16 (0.45)
	North Down	Pre	5	53 (49)	233 (263)	0.92 (0.24)	0.08 (0.06)	1.65 (1.22)
		Post	6	162 (128)	512 (147)	1.13 (1.14)	0.20 (0.19)	2.34 (0.35)

**Table 11:** Pre- vs. postrestoration statistical analyses of water quality at Haworth sites at baseflow and storm flow. To remove potential bias from pre- vs. postrestoration samples collected from different time periods, baseflow tests incorporated an equal number of samples from identical months in multiyear pre- and postrestoration periods (Apr., Jun., Jul., Sep., Oct., Nov., Dec., Jan., Feb., Mar., Apr., May, Jun., Jul., Aug., and Sep.). Storm flow tests incorporated all possible sampled storm events.

		North Up			North Down		
Flow	Parameter	Transform	*p*-Value	Notes	Transform	*p*-Value	Notes
Base	SRP	-	0.690	NS	-	0.184	NS
	TP	-	0.931	NS	-	0.396	NS
	NO_3_^−^	-	0.362	NS	sqrt	0.179	NS
	NH_3_	-	0.939	NS	log	0.926	NS
	TKN	-	0.739	NS	-	0.178	NS
	Turbidity	-	0.736	NS	-	0.548	NS
Storm	SRP	-	0.371	NS	*sqrt*	*0.058*	*post > pre*
	TP	-	0.850	NS	-	*0.053*	*post > pre*
	NO_3_^−^	sqrt	0.449	NS	-	0.688	NS
	NH_3_	-	0.197	NS	sqrt	0.165	NS
	TKN	-	0.857	NS	-	0.215	NS
	Turbidity	sqrt	0.872	NS	-	0.916	NS

## References

[1] Carpenter S.R., Caraco N.F., Correll D.L., Howarth R.W., Sharpley A.N., Smith V.H. (1998). Nonpoint pollution of surface waters with phosphorus and nitrogen. Ecol. Appl..

[2] Smith V.H., Tilman G.D., Nekola J.C. (1999). Eutrophication: Impacts of excess nutrient inputs on freshwater, marine, and terrestrial ecosystems. Environ. Pollut..

[3] Dodds W.K., Bouska W.W., Eitzmann J.L., Pilger T.J., Pitts K.L., Riley A.J., Schloesser J.T., Thornbrugh D.J. (2009). Eutrophication of US freshwaters: Analysis of potential economic damages. Environ. Sci. Technol..

[4] Scavia D., Allan J.D., Arend K.K., Bartell S., Beletsky D., Bosch N.S., Brandt S.B., Briland R.D., Daloğlu I., DePinto J.V. (2014). Assessing and addressing the re-eutrophication of Lake Erie: Central basin hypoxia. J. Great Lakes Res..

[5] Sharpley A., Jarvie H.P., Buda A., May L., Spears B., Kleinman P. (2013). Phosphorus legacy: Overcoming the effects of past management practices to mitigate future water quality impairment. J. Environ. Qual..

[6] Fales M., Dell R., Herbert M.E., Sowa S.P., Asher J., O’Neil G., Doran P.J., Wickerham B. (2016). Making the leap from science to implementation: Strategic agricultural conservation in Michigan’s Saginaw Bay watershed. J. Great Lakes Res..

[7] Sowa S.P., Herbert M., Mysorekar S., Annis G.M., Hall K., Nejadhashemi A.P., Woznicki S.A., Wang L., Doran P.J. (2016). How much conservation is enough? Defining implementation goals for healthy fish communities in agricultural rivers. J. Great Lakes Res..

[8] MWP (Macatawa Watershed Project) (2012). Macatawa Watershed Management Plan.

[9] Holden S. (2014). Monthly Water Quality Assessment of Lake Macatawa and Its Tributaries, April–September 2012.

[10] Walterhouse M. (1999). Total Maximum Daily Load for Phosphorus in Lake Macatawa.

[11] U.S. EPA (1993). Methods for Chemical Analysis of Inorganic Substances in Environmental Samples.

[12] APHA (1992). Standard Methods for Examination of Water and Wastewater.

[13] Dodds W.K., Smith V.H. (2016). Nitrogen, phosphorus, and eutrophication in streams. Inland Waters.

[14] Michalak A.M., Anderson E.J., Beletsky D., Boland S., Bosch N.S., Bridgeman T.B., Chaffin J.D., Cho K., Confesor R., Daloğlu I. (2013). Record-setting algal bloom in Lake Erie caused by agricultural and meteorological trends consistent with expected future conditions. Proc. Natl. Acad. Sci. USA.

[15] Schoumans O.F., Chardon W.J., Bechmann M.E., Gascuel-Odoux C., Hofman G., Kronvang B., Rubæk G.H., Ulen B., Dorioz J.M. (2014). Mitigation options to reduce phosphorus losses from the agricultural sector and improve surface water quality: A review. Sci. Total Environ..

[16] Kleinman P.J., Sharpley A.N., Withers P.J., Bergström L., Johnson L.T., Doody D.G. (2015). Implementing agricultural phosphorus science and management to combat eutrophication. Ambio.

[17] Lurling M., van Oosterhaut F., Faassen E. (2017). Eutrophication and warming boost cyanobacterial biomass and microcystins. Toxins.

[18] Smith D.R., King K.W., Johnson L., Francesconi W., Richards P., Baker D., Sharpley A.N. (2015). Surface runoff and tile drainage transport of phosphorus in the midwestern United States. J. Environ. Qual..

[19] Carpenter S.R., Booth E.G., Kucharik C.J. (2018). Extreme precipitation and phosphorus loads from two agricultural watersheds. Limnol. Oceanogr..

[20] Steinman A.D., Ogdahl M.E., Ruetz C.R. (2011). An environmental assessment of a small, shallow lake threatened by urbanization. Environ. Monit. Assess..

[21] Royer T.V., David M.B., Gentry L.E. (2006). Timing of riverine export of nitrate and phosphorus from agricultural watersheds in Illinois: Implications for reducing nutrient loading to the Mississippi River. Environ. Sci. Technol..

[22] Hanrahan B.R., Tank J.L., Christopher S.F., Mahl U.H., Trentman M.T., Royer T.V. (2018). Winter cover crops reduce nitrate loss in an agricultural watershed in the central US. Agric. Ecosyst. Environ..

[23] Mahl U.H., Tank J.L., Roley S.S., Davis R.T. (2015). Two-stage ditch floodplains enhance N-removal capacity and reduce turbidity and dissolved P in agricultural streams. J. Am. Water Resour. Assoc..

[24] Havens K.E., Jeppesen E. (2018). Ecological responses of lakes to climate change. Water.

[25] Loecke T.D., Burgin A.J., Riveros-Uregui D.A., Ward A.S., Thomas S.A., Davis C.A., St. Clair M.A. (2017). Weather whiplash in agricultural regions drives deterioration of water quality. Biogeochemistry.

[26] Steinman A.D., Chu X., Ogdahl M. (2009). Spatial and temporal variability of internal and external phosphorus loads in an urbanizing watershed. Aquat. Ecol..

[27] Steinman A.D., Hassett M.C., Oudsema M., Rediske R. (2018). Alum efficacy 11 years following treatment: Phosphorus and macroinvertebrates. Lake Reserv. Manag..

[28] Fuller L.M., Minnerick R.J. (2008). State and Regional Water-Quality Characteristics and Trophic Conditions of Michigan’s Inland Lakes, 2001–2005.

[29] Kelly P.T., González M.J., Renwick W.H., Vanni M.J. (2018). Increased light availability and nutrient cycling by fish provide resiliency against reversing eutrophication in an agriculturally impacted stream. Limnol. Oceanogr..

[30] Jarvie H.P., Sharpley A.N., Spears B., Buda A.R., May L., Kleinman P.J. (2013). Water quality remediation faces unprecedented challenges from “legacy phosphorus”. Environ. Sci. Technol..

[31] Clement D.R., Steinman A.D. (2017). Phosphorus loading and ecological impacts from agricultural tile drains in a west Michigan watershed. J. Great Lakes Res..

[32] Lam W.V., Macrae M.L., English M.C., O’Halloran I.P., Wang Y.T. (2016). Effects of tillage practices on phosphorus transport in tile drain effluent under sandy loam agricultural soils in Ontario, Canada. J. Great Lakes Res..

[33] Van Esbroeck C.J., Macrae M.L., Brunke R.I., McKague K. (2016). Annual and seasonal phosphorus export in surface runoff and tile drainage from agricultural fields with cold temperate climates. J. Great Lakes Res..

